# RNA interference in adult *Ascaris suum* – an opportunity for the development of a functional genomics platform that supports organism-, tissue- and cell-based biology in a nematode parasite

**DOI:** 10.1016/j.ijpara.2015.05.003

**Published:** 2015-09

**Authors:** Ciaran J. McCoy, Neil D. Warnock, Louise E. Atkinson, Erwan Atcheson, Richard J. Martin, Alan P. Robertson, Aaron G. Maule, Nikki J. Marks, Angela Mousley

**Affiliations:** a Parasitology, Institute for Global Food Security, School of Biological Sciences, Queen’s University Belfast, Belfast BT9 7BL, UK; b Department of Biomedical Sciences, Iowa State University, Ames, IA 50011, USA

**Keywords:** *Ascaris suum*, RNA interference, Nematode parasite, Reverse genetics

## Abstract

The sustainable control of animal parasitic nematodes requires the development of efficient functional genomics platforms to facilitate target validation and enhance anthelmintic discovery. Unfortunately, the utility of RNA interference (RNAi) for the validation of novel drug targets in nematode parasites remains problematic. *Ascaris suum* is an important veterinary parasite and a zoonotic pathogen. Here we show that adult *A. suum* is RNAi competent, and highlight the induction, spread and consistency of RNAi across multiple tissue types. This platform provides a new opportunity to undertake whole organism-, tissue- and cell-level gene function studies to enhance target validation processes for nematode parasites of veterinary/medical significance.

## Highlights

• RNA interference (RNAi) is readily achievable in adult *Ascaris suum.*
• RNAi is capable of spreading in adult *A. suum.*
• Multiple *A. suum* tissue-specific targets are susceptible to RNAi.
• Double-stranded RNA cocktails efficiently silence multiple targets simultaneously.
• RNAi is consistent across geographical isolates of *A. suum*.

## Graphical abstract

**Figure:**
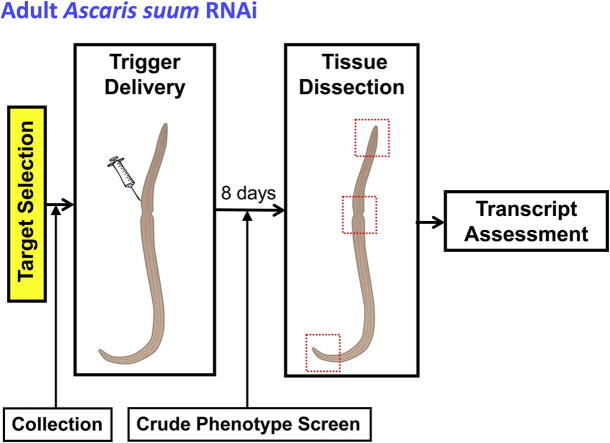

Nematode parasites are a major cause of disease in humans and animals where they undermine health and global food security. Over one billion people living in the developing areas of sub-Saharan Africa, Asia and the Americas are infected with at least one nematode parasite (see Lustigman et al., 2012), and the worldwide economic impact of nematode parasites to the livestock industry is estimated to be >GBP 10 billion per annum (Roeber et al., 2013). *Ascaris suum* is a gastrointestinal parasite of pigs which impacts significantly on pig health through reductions in finishing weights, feed conversion efficiencies and carcass quality. Recent publications have highlighted the zoonotic potential of *A. suum* and there is now evidence to suggest that *A. suum* is more than a close relative of the human pathogen, *Ascaris lumbricoides* (see Peng and Criscione, 2012). This is significant given that more than one billion people worldwide suffer from ascariasis and its resultant chronic morbidity (see Lustigman et al., 2012).

In the absence of viable, commercially available vaccines, the control of nematode parasites relies heavily on a small collection of chemotherapeutic drugs in both human and veterinary medicine (see Prichard et al., 2012). Significantly, multi-drug resistance has been reported in many of the key parasitic nematodes of livestock (see Kaplan, 2004) and drug resistance in human therapy is a rising concern (see Prichard et al., 2012).

Our future defence against nematode parasites in both humans and livestock is dependent on the identification and validation of novel chemotherapeutic targets. The availability of genomic and transcriptomic sequence data for nematodes is rapidly progressing; 25 published genomes and ⩾100 significant transcriptomic datasets are now available for mining (Blaxter and Koutsovoulos, 2014; Cotton et al., 2014; Foth et al., 2014; Jex et al., 2014; Rödelsperger et al., 2014; Tang et al., 2014). Genome-directed drug discovery has made significant inroads towards the identification of putative novel drug targets for nematode pathogens but validation will require the incorporation of functional tools in therapeutically relevant parasite species.

RNA interference (RNAi) is an appealing reverse genetics tool and, whilst its utility for the validation of drug targets in veterinary parasites is widely accepted, issues with their variable sensitivity to standard RNAi approaches have undermined the development of robust gene silencing platforms (see Maule et al., 2011). Although successful RNAi has been reported in ∼11 animal parasitic nematode (APN) species (see Maule et al., 2011; Tzelos et al., 2013), the utility of these data is hampered by a variety of species/tissue/target-specific inconsistencies in RNAi responsiveness. A key hurdle to the exploitation of putative targets identified in silico is the absence of validation tools that allow the elucidation of target function in relevant parasites.

Importantly, the adult stage of *A. suum* is well established as a parasite model system and more is known about the basic biology of *A. suum* than any other veterinary parasite. Amongst nematode parasites, *A. suum* has the most genomic/transcriptomic resources and it offers unrivalled utility for cell biology/physiology/biochemistry studies (Jex et al., 2011), supported by an extensive array of physiology- and cell-based assays for functional studies (Stretton and Maule, 2013). The tractability of *A. suum* as an experimental model makes the application of RNAi to the adult stage very appealing. To date, only larval stage *A. suum* have been shown to be RNAi susceptible (Islam et al., 2005; Xu et al., 2010; Chen et al., 2011).

Here we describe the development of an RNAi platform in adult *A. suum* that has the potential to significantly advance drug target validation in nematode parasites. Our data demonstrate that: (i) RNAi is readily achievable in adult *A. suum*. We have developed a method for the induction of RNAi in adult *A. suum* through the injection of double-stranded RNA (dsRNA) (100 μl; 200 ng/μl) into the pseudocoelomic cavity of female worms (see Fig. 1A). Adult *A. suum* were collected from the abattoir and transferred to the laboratory in mammal saline (0.9% NaCl). Worms were maintained in *Ascaris* Ringers Solution (ARS: 13.14 mM NaCl, 9.47 mM CaCl_2_, 7.83 mM MgCl_2_, 12.09 mM C_4_H_11_NO_3_/Tris, 99.96 mM NaC_2_H_3_O_2_, 19.64 mM KCl, pH 7.8) for a maximum of 24 h prior to use, and for the duration of the RNAi experiments (for specific methodology see Fig. 1).

We have achieved target transcript knockdown that: (a) is robust (e.g. ⩾60.0% (*P* < 0.05) knockdown across multiple targets (*A. suum* elongation factor 1a (*As-eft-*1); Elongation factor 1b (*As*-*eft*-2); GMP reductase (*As*-*gmpr*), troponin C (*As*-*tnc*-1), Ras-related protein (*As*-*rab*-3), haemoglobin (*As*-*hb*-1), and two nicotinic acetylcholine receptor subunits (*As-unc-*29 and *As-unc-*38)) at ⩾3 days post-RNAi trigger delivery; (b) occurs relatively quickly (significant transcript knockdown recorded as early as 24 h post-RNAi trigger delivery (e.g. 67.7 ± 4.4% knockdown of *As-eft*-1 in the tail region at 24 h, *P* < 0.05)); (c) is consistent and reproducible (100% success rate across eight targets with differential expression (see below)); and (d) persists (for up to 8 days; e.g. 98.5 ± 0.4% knockdown of *As-eft*-1 in the tail region at day 8) (see Figs. 1 and 2). Our findings are in contrast to the early literature describing RNAi susceptibility for some of the veterinary APNs including *Haemonchus contortus*, *Ostertagia ostertagi* and *Teladorsagia circumcincta* where hypervariability in the induction of gene silencing, that appeared to be largely target- and RNAi trigger delivery-dependent, was common (see Maule et al., 2011). One study has described the development of an RNAi platform for *H. contortus* through the investigation of different RNAi trigger delivery approaches across developmental stages and target types (Zawadzki et al., 2012), providing confidence in the application of RNAi in this species. In other APNs where inconsistencies were not highlighted (e.g. for *Nippostrongylus brasiliensis*, *Brugia malayi*, *Onchocerca volvulus*, *Trichostongylus colubriformis*, *A. suum*, *Litmosomoides sigmodontus*, *Heterorhabditis bacteriophora*, *Acanthocheilonema vitea*) the majority of studies only targeted one or two genes (see Maule et al., 2011; Tzelos et al., 2013), making it more difficult to assess the reliability of RNAi as a reverse genetics tool in these species.

(ii) RNAi is capable of spreading in adult *A. suum*. Our RNAi trigger delivery approach involves the injection of dsRNA into the pseudocoelomic cavity at a position approximately 1 cm anterior to the gonopore on the ventral side, where worms were injected at an angle (approximately 20°) to avoid piercing the gut (see Fig. 1A). We have recorded robust transcript knockdown for five targets in tissue segments distant from the site of injection (head and tail regions; see Figs. 1 and 2; *As*-*eft*-1, *As*-*eft*-2, *As*-*rab*-3, *As*-*tnc*-1, *As*-*gmpr; As-unc*-29, *As-unc*-38), and also in muscle bag cells (*As-unc-*29, *As-unc-*38; see Fig. 3) highlighting the spread of gene silencing triggers in adult *A. suum*. Note that the RNAi response in tissues adjacent to the injection site consistently lags behind that observed at the worm extremities across all time points (see Fig. 1B). The significance of the apparent lag is unclear. The fact that the temporal dynamics of the RNAi responses in the head, tail, gonopore and somatic muscle are similar enhances the interpretation of phenotypic readouts, and means that distinct tissues from the same worm can be used for a range of analyses such as transcript and protein quantitation and physiology/biochemical assays. This enhances the appeal of *A. suum* as a model nematode for reverse genetics.

The observed efficiency in adult *A. suum* RNAi is in contrast to that described for other APNs (see Maule et al., 2011). It seems likely that our injection of dsRNA directly into the pseudocoelom would enhance RNAi trigger delivery and could reduce variability in RNAi induction compared with that achieved in other APNs using alternative dsRNA delivery approaches (see Maule et al., 2011). Large adult worms contain ∼1 ml of pseudocoelomic fluid that is continuously circulated within the pseudocoelomic cavity by the activity of the somatic body wall muscle. This likely enables the efficient spread of the dsRNA within the pseudocoelomic fluid, bringing it into close contact with the various organs/tissues of the worm. This is likely to enhance the efficiency of RNAi in terms of both spread and direct access to cells.

It is interesting to note the absence of *sid*-1 and *sid*-2 homologues from *A. suum*, known to be important for the uptake and spread of environmental RNAi in nematodes, and the presence of an *rsd*-3 homologue, which is the most highly conserved of the proteins associated with the intercellular spread of RNAi (see Dalzell et al., 2011). The presence and/or absence of these proteins clearly does not limit RNAi capability in *A. suum* as is the case with other RNAi-competent nematode species which also lack these proteins (e.g. *Meloidogyne incognita* and *Globodera pallida*) (Dalzell et al., 2011; Cotton et al., 2014). As suggested by Dalzell et al. (2011), there may be uncharacterised RNAi pathway proteins that fulfil these roles.

(iii) Multiple *A. suum* tissue-specific targets are susceptible to RNAi including ‘neuronal’ genes. We have shown that a range of targets are susceptible to RNAi in adult *A. suum* including those expressed in: multiple tissues (*As-eft*-1; *As-eft*-2; *As-gmpr*); muscle (*As-tnc*-1); nerve (*As-rab*-3); gut/body wall (*As-hb*-1); and the neuromuscular system (*As-unc*-29, *As-unc*-38) (based on spatial expression of homologous genes in *Caenorhabditis elegans*) (see Figs. 1–3). Samarasinghe et al. (2011) suggest a correlation between target accessibility and RNAi susceptibility in that targets genes that are expressed in tissues accessible to an external source of dsRNA are more likely to be susceptible to RNAi. Here we do not see any tissue-specific differences in our ability to induce RNAi in adult *A. suum*. We believe that dsRNA delivery to the pseudocoel circumvents any potential RNAi trigger accessibility issues. The physiology of *A. suum* is such that the pseudocoelomic fluid perfuses multiple target tissues including the intestine, muscle (body wall, pharynx, ovijector, anus), nerve, hypodermis, and systems associated with reproduction and excretion, and it is therefore an ideal vehicle to carry dsRNA cargo to its target, offering a systemic delivery system.

Significantly, we have not noted any neuronal RNAi refractoriness that has been observed in other nematode species e.g*. C. elegans* (Timmons et al., 2001). Here we report significant silencing for three neuronal targets (*As*-*rab*-3, *As*-*unc*-29 and *As*-*unc*-38 (see Figs. 2 and 3)), providing confidence that neuronal targets are as susceptible to RNAi as those expressed in other tissues. Until now, successful RNAi of neuronal targets in parasitic nematodes has been restricted to the plant parasitic nematodes (see Maule et al., 2011).

(iv) A multi-target dsRNA cocktail delivery approach is efficient in inducing RNAi. Here we demonstrate that effective gene silencing is achievable when two dsRNA species, targeting different transcripts (e.g. *As-unc*-29 and *As-unc*-38) are delivered as a cocktail (Fig. 3). This is significant with respect to experimental set up as it reduces time, cost and worm/tissue requirements. In addition, from a gene function perspective, this approach enables the co-silencing of multiple related genes where functional redundancy may be a concern, but promiscuous dsRNA design is not possible. It will be interesting to determine the limitations of this approach with respect to RNAi efficiency, and to assess the efficacy of small interfering RNA (siRNA)-mediated RNAi in *A. suum*.

(v) RNAi is consistent across geographical isolates of *A. suum*. Here we present data to show that *A. suum* from different geographical regions were equally susceptible to RNAi (Fig. 3), and that there was no significant difference in the dynamics associated with the induction of knockdown across geographical isolates. These data are significant given that different strains of *C. elegans* appear to be variably susceptible to somatic RNAi (Félix et al., 2011), and emphasise the potential intercontinental utility of RNAi in adult *A. suum*, supporting its widespread application.

We believe that this study is the first report of successful gene silencing in adult *A. suum*. We have shown that RNAi is achievable across multiple targets and tissues and present a novel, efficient RNAi delivery approach. The appeal of the *A. suum* RNAi platform described here is enhanced by the unique experimental tractability of the adult worm and the availability of a number of post-RNAi functional bioassays at the whole worm (body waveform, egg output, development), tissue (body wall muscle, pharynx, ovijector) and cell (muscle and neuron electrophysiology) levels (see Pecson et al., 2006; Stretton and Maule, 2013). This provides an appealing platform for the identification and validation of drug targets in parasitic nematodes of veterinary and human importance.

## Figures and Tables

**Fig. 1:**
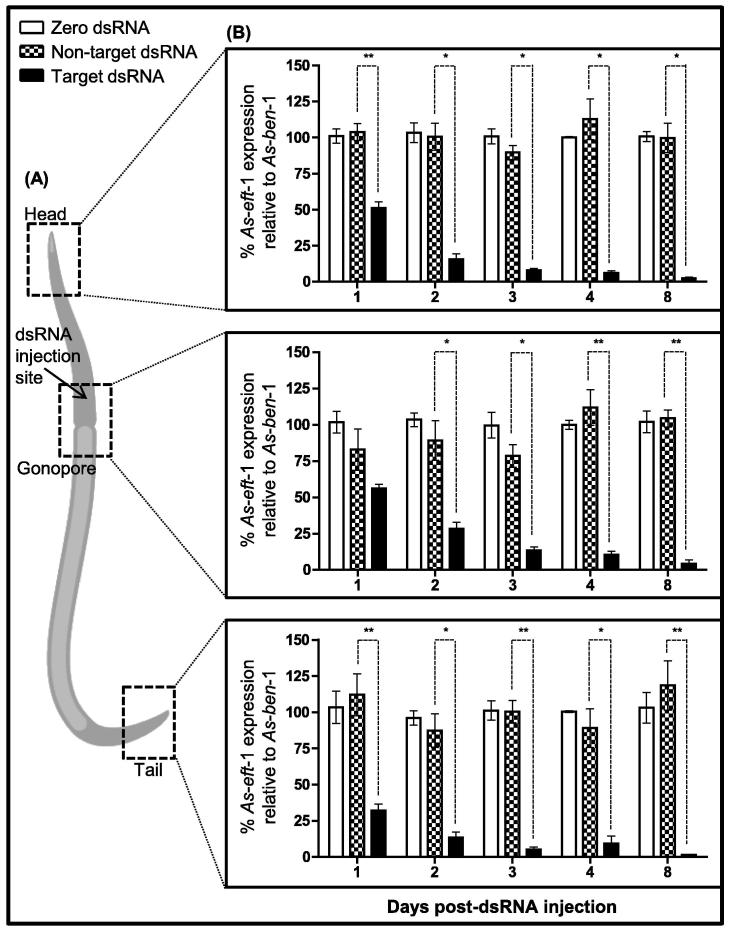
A novel adult *Ascaris suum* RNA interference (RNAi) trigger delivery approach induces a robust systemic RNAi response in distinct tissue regions. (A) Novel RNAi trigger delivery approach: double-stranded RNA (dsRNA) was injected anterior to the gonopore and transcript levels were assessed in head, gonopore and tail tissue segments. (B) *A. suum* translational elongation factor 1a (*As*-*eft*-1) target transcript knockdown was significant (>95% by day 8, *P* < 0.05), and persistent (up to 8 days) in tissues remote from the RNAi trigger delivery site. Head region: *As*-*eft*-1 knockdown in target (*As*-*eft*-1) dsRNA treated worms compared with zero dsRNA controls is 48.8%, 84.4%, 92%, 94.1% and 97.6% at 1, 2, 3, 4 and 8 days post-injection, respectively (*n* = 6 for all time points except day 4 where *n* = 4); gonopore region: *As*-*eft*-1 knockdown in target dsRNA treated worms compared with zero dsRNA controls is 64%, 71.7%, 86.5%, 89.6% and 95.7% at 1, 2, 3, 4 and 8 days post-injection, respectively (*n* = 6); tail region: *As*-*eft*-1 knockdown in target dsRNA treated worms compared with zero dsRNA controls is 67.8%, 86.7%, 94.8%, 90.8% and 98.5% at 1, 2, 3, 4 and 8 days post-injection, respectively (*n* = 6 for all time points except day 4 where *n* = 4). Note that *A. suum* elongation factor 1b (*As*-*eft*-2) knockdown (head: 87.7 ± 2.8% (*n* = 6; *P* < 0.05, target dsRNA versus non-target dsRNA); gonopore: 81.1 ± 10.4% (*n* = 6; *P* < 0.05, target dsRNA versus non-target dsRNA); tail: 96.7 ± 1.2% (*n* = 6; *P* < 0.05, target dsRNA versus non-target dsRNA)) was assessed in *As*-*eft*-1-dsRNA treated worms compared with zero dsRNA control worms, 8 days post-injection (graph not shown); error bars represent S.E.M.; ^*^P < 0.05, ^**^P < 0.01. Methods were as follows: (i) dsRNA synthesis. Target and control T7-labelled PCR products (target and control GenBank accession numbers, sense (S) and antisense (AS) primers (5′-3′), and amplicon sizes were: *As-eft*-1 (Elongation factor 1a; accession No. BK006061), S 5′-GAGGCTCTGTCGCTTCTGAC-3′, AS 5′-TTGAGGAACTTCGGGTTGTC-3′ (219 bp); *As-eft*-2 (Elongation factor 1b; accession No. JI172981) S 5′-AATGAACCACCCGGGACAGA-3′, AS 5′-TCAACGCACAGCGGTTTTGT-3′ (201 bp); *As-gmpr* (GMP reductase; accession No. JO467845), S 5′-ATCTTTGCGGGCAATGTCGT-3′, AS 5′-TGCCCGTTGAGACCATGAGA-3′ (185 bp); *As-tnc*-1 (troponin C; accession No. JO472030), S 5′-CGACGAGTTTTGCGCTCTTG-3′, AS 5′-TCCACAGCCGCTTCTAGCTG-3′ (192 bp); *As-rab*-3 (synaptic vesicle protein; accession No. JI178604), S 5′-CCGATCAGCTCGGTTTGGAG-3′, AS 5′-TGCACTGTTGTGATGGTGGTTT-3′ (195 bp); *As-hb-*1 (haemoglobin; accession No. AAA29374), S 5′-TCTTTGCGCCACCTACGATG-3′, AS 5′-ACTCCCTGCCGATTTCATGC-3′ (197 bp); *As-unc*-29 (nicotinic acetylcholine receptor subunit; accession No. ADY42984), S 5′-ACTTTCGGGGACCGACCACT-3′, AS 5′-GTGATGTGCGGACGGATTCA-3′ (209 bp); *As-unc*-38 (nicotinic acetylcholine receptor subunit; accession No. ERG87157), S 5′-TGGTGGTGTCAGCGTGCTTT-3′, AS 5′-TGCTCATCGAATGGAAACCATC-3′ (204 bp); non-target control dsRNA (Neomycin Phosphotransferase; accession No. U55762), S 5′-GGTGGAGAGGCTATTCGGCT-3′, AS 5′-CCTTCCCGCTTCAGTGACAA-3′ (223 bp); note that dsRNA T7 template primers had a T7 promoter (5′-TAATACGACTCACTATAGGG-3′) appended to the 5′ end) with sense and antisense polarity were amplified (cycling conditions: 95 °C × 5 min, 40 × (95 °C × 30 s, 60 °C × 30 s, 72 °C × 2 min), 72 °C × 10 min) from sequence-verified cDNA templates (GATC Biotech, Germany, http://www.gatc-biotech.com), and used to synthesise dsRNA with either the MEGAshortscript™ Kit (Life Technologies, USA), or T7 RiboMAX™ Express RNAi kit (Promega, USA) according to manufacturer’s instructions. dsRNA was eluted in *Ascaris* saline (125 mM CH3COONa, 24.5 mM KCl, 4 mM NaCl, 11.8 mM CaCl2, 9.8 mM MgCl2, 5 mM MOPS buffer, pH 6.8), and visualised on a 1.2% agarose gel to confirm integrity. Concentration and purity were assessed using a Nanodrop ND-1000-A spectrophotometer (Labtech, UK). (ii) dsRNA delivery and tissue dissection. Adult female *A. suum* (⩾15 cm) were injected (26 gauge needle) with 100 μl of dsRNA (200 ng/μl) or *Ascaris* saline (zero dsRNA control), into the pseudocoelomic cavity (1 cm anterior to the gonopore on the ventral side of the worm). dsRNA and *Ascaris* saline was supplemented with 1% green food dye to facilitate visualisation of pseudocoelomic delivery. *Ascaris suum* were maintained in *Ascaris* Ringer Solution (13.14 mM NaCl, 9.47 mM CaCl_2_, 7.83 mM MgCl_2_, 12.09 mM C_4_H_11_NO_3_/Tris, 99.96 mM NaC_2_H_3_O_2_, 19.64 mM KCl, pH 7.8) for 1–8 days post-injection after which tissue sections from the head (1.5 cm segment), tail (1.5 cm segment) and gonopore regions (1 cm segment) were dissected, snap-frozen in liquid nitrogen and stored at −80 °C until use. Where relevant, muscle bag cells were dissected as a tissue layer from a body wall flap (1 cm long; including both dorsal and ventral tissue), that was excised ∼2 cm anterior to the gonopore. Post-dissection, muscle bag cell layers were submerged in autoclaved *Ascaris* perienteric fluid (APF: 23 mM NaCl, 110 mM NaC_2_H_3_O_2_, 24 mM KCl, 6 mM CaCl_2_, 5 mM MgCl_2_, 11 mM C_6_H_12_O_6_, 5 mM C_8_H_18_N_2_O_4_S, pH 7.6). Sterile scissors were used to snip the arm region of the muscle tissue and release the layer of muscle bag cells from the rest of the body wall. Note that both ventral and dorsal muscle bag cell layers were dissected from the same body wall flap and processed together. (iii) Post-RNAi transcript analysis. Individual tissue segments were ground to a fine powder whilst frozen using a mortar and pestle. Total RNA was extracted using TRIzol® Reagent (Life Technologies), treated with DNase I (Ambion TURBO DNase, Life Technologies), standardised to 1800 ng/μl, and used for cDNA synthesis (Applied Biosystems High Capacity RNA-to-cDNA reverse transcription kit (Life Technologies) according to manufacturer’s instructions). Target and reference gene (*As-ben*-1) transcripts were amplified from each cDNA in triplicate by quantitative real-time PCR (qPCR) using a Qiagen Rotor-Gene 5-plex HRM, and FastStart SYBR Green Master (Roche, Switzerland) (target and reference gene S- and AS primers, and amplicon sizes were as follows: *As-eft*-1, S 5′-ATTCTCCGAGACTCGCTTCA-3′, AS 5′-GAGATCGGCACGAAAGCTAC-3′ (99 bp); *As-eft*-2, S 5′-GTCCCACCACAGAGGCCAAC-3′, AS 5′-CACGACCCACTGGCACTGTC-3′ (94 bp); *As-gmpr*, S 5′-GAGTGGAAGGCTTTCGTGCAA-3′, AS 5′-CCGATGTCGATATTCCCGATG-3′ (85 bp); *As-tnc*-1, S 5′-TCGATGGATCGCAAATCGAA-3′, AS 5′-TGGGTGGCCATGATGTAACC-3′ (82 bp); *As-rab*-3, S 5′-CGCGGGGATAAACGTGTCA-3′, AS 5′-GAAGCCCATTGCACCACGAT-3′ (105 bp); *As-hb-*1, S 5′-GCTTTCAAGGACCGCGAGAA-3′, AS 5′-GCAGATGACAGGCGAGCAGA-3′ (97 bp); *As-unc*-29, S 5′-CCCCACTGATTCAGGCGAAA-3′, AS 5′-TGGGTGGCAGGATCTTCGAT-3′ (95 bp); *As-unc*-38, S 5′-GGCCACTTGCTGATACCGATG-3′, AS 5′-GGCTTACGCGATGTTCGTGA-3′ (102 bp); *As-ben*-1 (beta-tubulin; GenBank accession number FE913811), S 5′-CCCACATACGGAGACCTCAACC-3′, AS 5′-CCAATTTGCGCAAGTCTGCAT-3′ (103 bp); cycling conditions: 9 °C × 10 min, 40 × (95 °C × 15 s, 60 °C × 15 5 s, 72 °C × 30 s), 72 °C x10 min). PCR efficiencies were calculated using Real-time PCR Miner (http://www.miner.ewindup.info/index.htm) and used for relative quantification of target gene transcript levels by the augmented comparative ΔΔCt method (Pfaffl, 2001). Changes in target gene transcript abundance were analysed using the non-parametric Kurskal–Wallis test, and the Dunn’s post-test (to assess the significance of the zero dsRNA and target dsRNA treatments against the non-target dsRNA, separately; note that we did not detect a significant difference between zero dsRNA and non-target dsRNA controls in any dataset) with GraphPad PRISM Version 5 (GraphPad Software, Inc., USA).

**Fig. 2:**
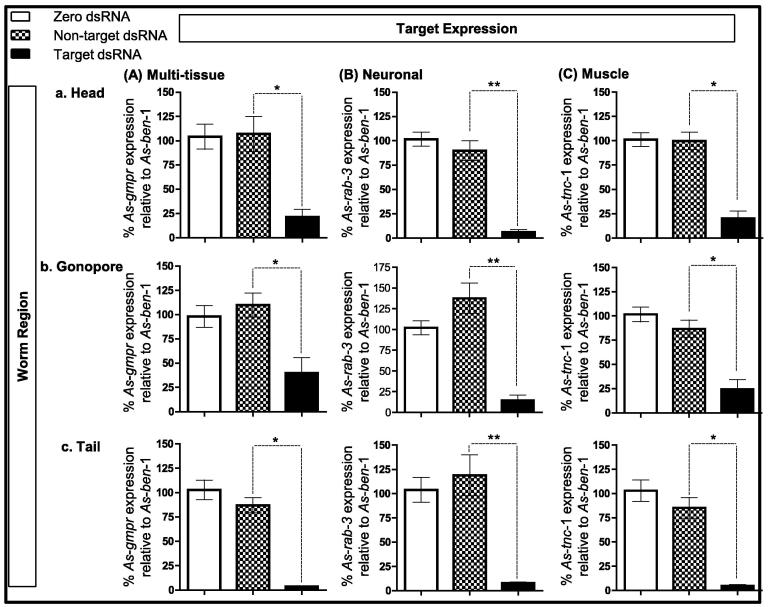
Targets with differential expression patterns are susceptible to RNA interference (RNAi) in adult *Ascaris suum*, including those restricted to one tissue type. (A–C) Transcript reduction in a range of target tissues across head (a), gonopore (b) and tail (c) regions 3 days post-injection, including those with: (A) multi-tissue expression: GMP reductase (*As*-*gmpr*) knockdown (Aa) head: 78.5 ± 8.0%; (Ab) gonopore: 60.0 ± 15.7%; (Ac) tail: 96.4 ± 0.1%) in *As*-*gmpr* double-stranded RNA (dsRNA)-treated worms compared with zero dsRNA control worms (*n* = 6). (B) Neuronal expression: *A. suum* synaptic vesicle protein (*As*-*rab*-3) knockdown (Ba) head: 93.8 ± 2.5%; (Bb) gonopore: 85.4 ± 6.4%; (Bc) tail: 95.2 ± 1.4%) in *As*-*rab*-3-dsRNA treated worms compared with zero dsRNA control worms (*n* = 6); (C) Muscle eExpression: *A. suum* troponin C (*As*-*tnc*-1) knockdown (Ca) head: 80 ± 7.6%; (Cb) gonopore: 75.6 ± 9.8%; (Cc) tail: 92.1 ± 1.2%) in *As*-*tnc*-1-dsRNA-treated worms compared with zero dsRNA control worms (*n* = 6); in addition, knockdown was achieved for a gut and body wall expressed target (haemoglobin (*As-hb*-1; gonopore: 74.2 ± 11.2%; day 3, *n* = 3; *P* < 0.05, target dsRNA versus non-target dsRNA); graph not shown) and two nicotinic acetylcholine receptor subunits expressed in the neuromuscular system (*As-unc*-29, *As-unc*-38; see Fig. 3). Error bars represent S.E.M.; ^*^P < 0.05, ^**^P < 0.01.

**Fig. 3:**
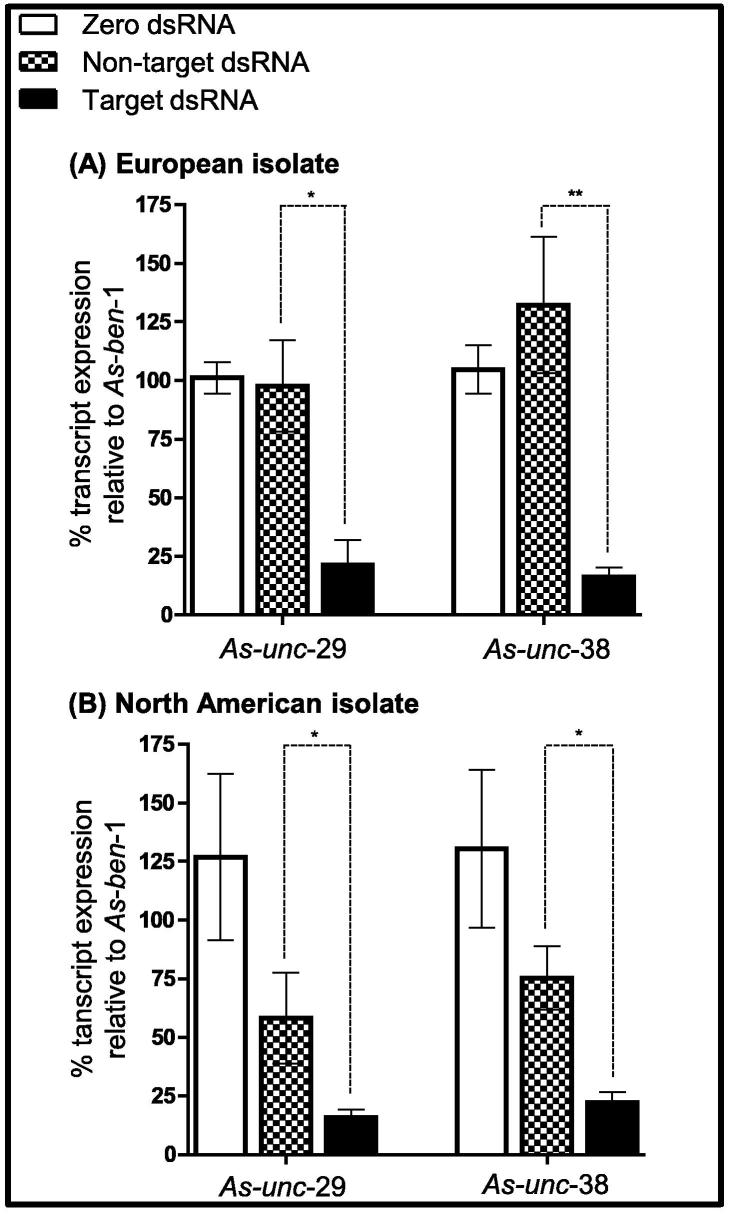
RNA interference (RNAi) is achievable using a ‘multi-target dsRNA cocktail’ delivery approach and in different geographical isolates of *Ascaris suum.* (A) Two nicotinic acetylcholine receptor subunits, *As-unc*-29 and *As-unc*-38, transcript knockdown (8 days post injection) in gonopore-tissue segments of adult *A. suum* collected in Ballymena, Northern Ireland (European Isolate: *As*-*unc*-29 knockdown in target dsRNA-treated worms compared with zero dsRNA controls is 92.9 ± 1.8% (*n* = 6); *As*-*unc*-38 knockdown in target dsRNA-treated worms compared with zero dsRNA controls is 83.9 ± 3.3% (*n* = 6)). (B) *As-unc*-29 and *As-unc*-38 transcript knockdown (8 days post-injection) in gonopore tissue segments of adult *A. suum* collected in Marshalltown, Iowa, U.S.A. (North American Isolate: *As-unc-*29 knockdown in target dsRNA-treated worms compared with zero dsRNA controls is 84.2 ± 3.4% (*n* = 6); *As-unc-*38 knockdown in target dsRNA-treated worms compared with zero dsRNA controls is 77.1 ± 4.5% (*n* = 6)). Note that transcript knockdown was also assessed in the head region (European isolate only: *As*-*unc*-29 knockdown in target dsRNA-treated worms compared with zero dsRNA controls is 86.3 ± 7.7% (*n* = 5); *P* < 0.05, target dsRNA versus non-target dsRNA; *As*-*unc*-38 knockdown in target dsRNA-treated worms compared with zero dsRNA controls is 83.9 ± 7.4% (*n* = 5); *P* < 0.05, target dsRNA versus non-target dsRNA; graphs not shown), and muscle bag cells (North American isolate only: *As*-*unc-*29 knockdown in target dsRNA-treated worms compared with zero dsRNA controls is 82.2 ± 6.2% (*n* = 6); *P* < 0.05, target dsRNA versus non-target dsRNA; *As*-*unc*-38 knockdown in target dsRNA-treated worms compared with zero dsRNA controls is 85.6 ± 3.8% (*n* = 6); *P* < 0.05, target dsRNA versus non-target dsRNA; graphs not shown). Error bars represent S.E.M; ^*^P < 0.05, ^**^P < 0.01. Note that in all cases target dsRNA-treated worms were injected with a cocktail of dsRNA-*As-unc*-29 and dsRNA-*As-unc*-38.
